# Using a multi-module web-app to prevent substance use among students at a Hispanic Serving Institution: development and evaluation design

**DOI:** 10.1186/s12889-022-13428-x

**Published:** 2022-06-15

**Authors:** Bethany K. W. Rainisch, Linn Dahlman, Jorge Vigil, Myriam Forster

**Affiliations:** Department of Health Sciences, California State University, Northridge, 18111 Nordhoff St., Northridge, CA 91330 USA

**Keywords:** mHealth, Substance use, College students, App, Intervention

## Abstract

**Background:**

Despite effective strategies to prevent substance use and substance use disorders among college students, challenges remain. As young adults’ preference for and comfortability with web-based technology continues to increase, leveraging innovative approaches to rapidly evolving mHealth technology is critical for the success of lowering the risk for substance use and related consequences in college populations, and especially those at Hispanic Serving Institutions. Therefore, the present study describes the rationale, development, and design of iSTART, a novel web-app to prevent substance use among students.

**Methods:**

The web-app was developed following the intervention mapping protocol, and in collaboration with numerous stakeholders, including a community-based partner specializing in substance abuse prevention and treatment. A 30-day multi-module web-app intervention was developed based on key theoretical constructs, behavior change strategies, and practical module components: attitudes (knowledge), perceived susceptibility (risk perceptions), subjective norms (normative re-education), and self-efficacy (refusal skills). This intervention will be evaluated via a time series design using a sample of 600 students randomly assigned to either the intervention, comparison, or control condition at a public institution in southern California.

**Discussion:**

The iSTART web-app is an innovative and sustainable program ideal for college campuses with diverse student populations. If this prevention web-app is successful, it will significantly contribute to the evidence of effective substance use interventions in the college setting, and identify the benefits of mHealth programs to prevent future substance use.

**Trial registration:**

NCT05362357 retrospectively registered on May 4, 2022 on clinicaltrials.gov.

## Background

Although the college years are a period of exploration and positive transformation, college students are at high risk for health-compromising behaviors such as alcohol and drug use. In 2020, approximately one-third of U.S. college students reported past-month binge drinking, 43% used marijuana, 16% used nicotine vaping devices, and 3% misused prescription drugs [1]. The college years are a stressful period of transition when young adults experience many changes in emotional, social, and identity development – processes that can increase risky behaviors, especially substance use. Use of alcohol, marijuana, and other drugs can contribute to adverse health and social outcomes and threaten students’ ability to successfully obtain a degree, develop enduring social bonds, and participate in the workforce [2–4]; domains of functioning that promote healthy outcomes over the life course [5, 6]. Relatedly, nearly 10% of students report their alcohol use has led to health, social, legal, or financial problems at least once [1]; while over 500,000 students are injured or assaulted, 8.5% are arrested, and approximately 2% attempt suicide every year due to their own or others' substance use [7, 8]. College students and young adults who report past 30-day polysubstance use, such as using both alcohol and marijuana (or other vaping device), or prescription medication (e.g., opioids) in conjunction with alcohol [9], are at especially high risk for major depressive episodes or serious mental illness [10].

Research suggests the negative social, legal, and health-related outcomes linked to substance use may disproportionately affect some populations more than others. While non-Hispanic White young adults report the highest rate of overall alcohol consumption, Hispanic adults are more likely to have higher rates of alcohol dependence, and consume five or more drinks in one sitting than their non-Hispanic White peers [11, 12]. Moreover, the 2006 Hispanic Americans Baseline Alcohol Survey (HABLAS) indicated nearly a quarter of participants reported two or more alcohol-related problems [13]. In regards to opioids, from 2014 to 2017 across all race/ethnic groups, Hispanics had the second highest deaths resulting from synthetic opioids, increasing 617% [14]. In 2019, 5.7% of Hispanics age 18–24 reported misusing opioids or prescription pain meds, 48% reported past month alcohol use, and 20% reported using marijuana in the last 30 days [15]. Such college student data and specifically young adult Hispanic substance use and negative outcomes, underscores the importance of culturally relevant substance use prevention programs on college campuses that can target this at-risk population.

In response to the growing concern of young adult and college student substance use, the National Institute of Alcohol Abuse and Alcoholism (NIAAA) promotes and facilitates the adoption of evidence-based research focusing on individual-level alcohol intervention strategies implemented on college campuses [16], and the Substance Abuse and Mental Health Services Administration (SAMHSA) identifies evidence-based strategies and programs to prevent substance misuse among those 18–25 years old [17]. Current college substance use intervention and prevention programs often utilize traditional face-to-face or online programs to reduce alcohol consumption [16]. Among the highly effective are individual-level strategies, such as brief motivational interventions (BMI) and the Brief Alcohol Screening and Intervention for College Students (BASICS), a face-to-face harm reduction approach incorporating self-management strategies to reduce drinking [18, 19]. Similarly, fully computerized campus E-interventions, especially those that incorporate online alcohol screening and automated personalized feedback, are proving efficacious [20, 21]. AlcoholEdu for College, an online program with knowledge-based quizzes and personalized feedback, positively impacts college alcohol-related attitudes, behaviors, and consequences [22], and has shown short-term effects among freshmen. However, nearly all reported data are from colleges with predominantly non-Hispanic White student bodies [23], and are focused solely on alcohol use. Although face-to-face settings or static web-based methods have proven successful in reducing college alcohol use, in-person settings and current antiquated platforms are limited in scope and versatility, and fail to address polysubstance use among diverse college student bodies.

Currently, only three of the nearly two dozen evidence-based individual-level alcohol and substance use intervention strategies listed by NIAAA are identified as highly effective and low cost: normative re-education, skills training, and personalized feedback (e.g., eCHECKUP TO GO) [20]. Additionally, no programs in SAMHSA’s list of evidence-based programs in college settings to prevent substance misuse among those 18–25 years old include substances beyond alcohol [17]. Some college-based interventions incorporate brief motivational interviewing (e.g., BASICS), that while effective [24, 25], are costlier, targeted toward risky substance users (predominantly alcohol), and generally require face-to-face student interaction [26]. While research has identified several essential ingredients to substance use prevention, few campus prevention efforts have leveraged mHealth delivery systems or web-app technology; and no comprehensive substance use prevention efforts using this medium have been broadly tested [27].

Despite significant progress leading to the identification of modifiable risk and protective factors and effective strategies to prevent substance use and substance use disorders, challenges remain. Evidence-based practices are not being broadly adapted and sustained, and emerging drug trends (e.g., the opioid epidemic, changing policies related to cannabis, vaping/e-cigarettes) present new challenges for prevention research and practice [28–30]. One of the biggest barriers for prevention work with college populations is how to attract and reach student populations. The number of young adults using smart devices to connect with diverse social and didactic networks is increasing [31], as well as their preference for and comfortability with web-based technology. As such, leveraging innovative approaches to rapidly evolving mHealth technology for prevention programming is critical for the success of public health efforts aimed at lowering the risk for substance use and related consequences in college populations, and especially those at Hispanic Serving Institutions.

There is a strong link between Healthy People (HP) 2030’s focus on reducing substance use among young people and the need to develop, test, scale, and sustain evidence-based programs. Especially programs designed to limit engagement in health-compromising behaviors, such as substance use; and prevent progression to risky use in service or educational systems. Importantly, HP 2030 identified the following primary public health objectives: reduce a) the number of adults who use marijuana daily or almost daily, b) the proportion of adults who use drugs in the last month, c) the number of people who misuse prescription drugs and, d) the number of young adults who binge drink; goals this study aims to address [32]. This paper describes the rationale, intervention design, and evaluation of the web-app iSTART (initiative for Services in Tech-Health and Rapid Testing) to prevent substance use (i.e., alcohol, marijuana, nicotine, prescription drugs, and illicit drugs) among college students at a Hispanic Serving Institution. We hypothesize that the intervention will prevent increases, limit, or lower substance use (i.e., measured by baseline, 30-day and 90-day binge drinking, marijuana use, etc.) compared to comparison and control groups. We anticipate that the increases in 1) substance-related knowledge, 2) perceived health risk associated with substance use, 3) confidence in refusal skills, and 4) correcting perceptions of substance use prevalence are the mechanisms that reduce and limit substance use behaviors of students in the prevention condition.

## Methods

### Study design and setting

The design consists of participant recruitment and randomization of participants to either intervention, comparison, or control conditions with a time series evaluation design. The intervention will occur at a large Hispanic Serving public Institution in southern California. The students in the intervention group will receive a 30-day web-app consisting of five substance-specific modules, while the students in the comparison group will receive a single abbreviated module, and those in the control group will only have access to standard online University substance abuse resources. Study data collection periods consist of a baseline survey after recruitment and assignment to condition, an exit survey after completing the 30-day iSTART web-app intervention (or 30-day comparison or control), and a 90-day follow-up survey.

### Study population and recruitment

The study population will consist of college students age 18–30. We will recruit 200 students in the prevention condition, 200 students in the comparison group condition, and 200 students in the matched control condition. We will use mixed models with a random effect for individual to test for the difference in longitudinal slopes with days drinking and marijuana use (example outcomes). We assume an average of 3 measurements per person and a correlation of 0.10 among intra-individual measurements, using a 2-sided test with alpha = 0.05 and 80% power, we will be able to detect a difference in frequency of alcohol and marijuana use at the final endpoint for prevention vs. control and for prevention vs. comparison group with Cohen’s d effect sizes of approximately 0.20 and 0.23, respectively, meaning we will be able to detect small minimum effect sizes among groups. Power analysis indicate that to detect a small effect size, adjusted for covariates across groups (all three groups: control, comparison, and prevention intervention), a minimum of 525 participants are needed over three years. An oversampling of 15% will be conducted to account for possible attrition across surveys. A total sample of 690 college students (evenly randomized to control, comparison, and intervention groups) will be recruited over three years to achieve sufficient statistical power.

Recruitment of student participants will include printed flyers with study information available on campus during tabling events for students at the beginning of the academic term, during campus events (both in-person and virtual) with different campus organizations; posters on lawn signs, campus buildings, and businesses; and electronic posting on digital campus billboards. Students will be invited to participate via email recruitment fliers to Student Housing and through college and department announcements. All recruitment materials will include the intervention website information, where students can review the study requirements, check their eligibility, and enroll in the study. Initial screening of participants includes an electronic review of study objectives, completion of a brief questionnaire for alcohol use disorder (AUD) or substance use disorder (SUD) risk [based on the Diagnostic and Statistical Manual of Mental Disorders, Fifth Edition (DSM-5) criteria], and electronic consent. Exclusion criteria include: individuals who are not college students, under 18 or over 30 years of age, or at high risk for AUD or SUD. Students screened as high risk for AUD or SUD will be referred to additional resources and excluded from the study.

The study adheres to the Belmont Report Ethical Principles and Guidelines for the Protection of Human Subjects of Research. Approval for the intervention study was provided by the Institutional Review Board.

### Intervention development

The intervention web-application consists of five weekly substance-specific modules that provide prevention information geared toward college students. The brief 15-minute web-app modules emphasize simplicity, interactivity, and accessibility. The development of each module’s prevention-related content is guided by evidence-based strategies. The intervention mapping protocol and its collaborative principles were used to develop the web-app [33], in addition to existing needs assessment data from the National College Health Assessment (NCHA) [34], and prior feedback from a pilot Telehealth alcohol program [35].

Diverse stakeholders were engaged following the examination of needs assessment data and the intervention mapping protocol. To develop the content of the intervention, a partnership was formed with a local community-based organization (CBO) specializing in substance abuse prevention and treatment services. Alcohol, tobacco, and other drugs (ATOD) prevention specialists at the CBO created preliminary prevention messages for each substance-specific module including substance-related facts, health risk information, and safe use guidelines from numerous reliable substance abuse sources such as: NIAAA, Centers for Disease Control and Prevention (CDC), American Addiction Centers (Drugabuse.com), National Institutes of Drug Abuse (NIDA), drugabuse.gov, U.S. Food and Drug Administration (FDA), truth initiative, and Generation Rx. A licensed psychologist created short self-assessments for each substance module and developed corresponding feedback. Other stakeholders (i.e., public health Faculty and students, campus recreational center, clubs, campus counseling services) were frequently consulted to provide insight on recommendations for healthy coping strategies/healthy alternative activities, suggestions for improving social wellbeing, and related campus resources accessible to students.

To improve user engagement and information retention, content was delivered using health literacy and health communication strategies [36], such as easy-to-read material, shorter statements, bulleted lists or checklists, and culturally and age-relevant language. Purposeful and relatable photos and graphics were retrieved from open-source platforms such as Creative Commons, Pixabay, and Pexels. Videos that were not created by faculty and students, were sourced from other organizations that allowed for free use, such as Generation RX and ReachOut.com [37, 38]. Content that aims to correct student perceptions of the prevalence of use utilized campus substance use data as reported in the most recent National College Health Assessment [34].

To develop the technical aspects of the web-application, a campus-based web-developer was contracted. During the creation process, web developers met weekly with the study team to discuss design, in-app features, randomization algorithms, and user reminders. The app developers received intended module content and provided mockups to present a visual profile with interactive elements (i.e., quizzes, flip cards, carousels, and videos). They ensured all university accessibility requirements were met, and content was appropriately designed for diverse smart device viewing, usable from either a mobile phone, tablet, or laptop/computer. The application is hosted on a university server and leverages the already existing institutional identification management system, which ensures that participants use their university username and log-in, providing security and confidentiality.

### Theoretical basis

Health behavior theory constructs such as self-efficacy [39] and perceived susceptibility [40], attitudes and subjective norms [41], in combination with individual-level evidence-based substance use strategies including: normative re-education [42], personalized feedback [43, 44], goal setting, and protective strategies [45, 46] are the foundation of the web-app intervention. The substance use prevention modules foster knowledge regarding the health risks associated with substance misuse, perceptions of the prevalence of use, and empower students through refusal skills [47, 48], increase perceived awareness of risks, and describe and outline substance-related coping skills for students to navigate the challenges of college life.

### Intervention modules

The 30-day prevention web-app consists of five weekly substance-specific modules: alcohol, marijuana, nicotine, prescription drugs, and illicit drugs. Each module contains a similar pattern of elements to address the aforementioned health behavior constructs, utilizing evidence-based behavior change strategies and interactive practical applications (see Table 1).

**Table 1:** Intervention framework: theoretical constructs, evidence-based behavior change strategies, practical application, and specific outcome measures

Theoretical constructs	Behavior change strategies	Practical application	Outcome measures
**Attitudes/Knowledge**	**Educational content**	- Identify potency, present legal facts via flip cards, quizzlets, videos, etc.	*- Substance-specific knowledge (4 items)* *- Do you ever feel bad about your alcohol or drug use?*
**Perceived susceptibility**	**Correct risk perceptions**	- Define what constitutes risky substance use. - Present health risks of substance misuse and dependency risk via flip cards, quizzlets, videos, etc.	*- What level of risk do you think people have of harming themselves physically or in other ways when they [use a substance]? (6 items)*
**Subjective norms**	**Normative re-education**	- Guess percentage of peer use on campus; show actual vs. perceived use via pie charts. - Inform how college substance use is less frequent than perceived.	*- In the last 30 days, what percent of students do you think used [substance]? (14 items)*
**Self-efficacy**	**Refusal skills**	- Examples of how to say “no” in a variety of scenarios.	*- I would be able to say no if a friend offered me [substance]. (2 items)*
	**Personalized feedback**	- Self-assessment and individualized feedback.	
	**Skills/knowledge for responsible use**	- Responsible use checklist.	
	**Protective strategies**	- Identify healthy alternative activities and support (physical, mind-body, social). - Checklist of healthy study habits. - Links to virtual and on-campus support resources.	
	**Goal setting**	- Write SMART goal for healthy strategies	

**Fig. 1:**
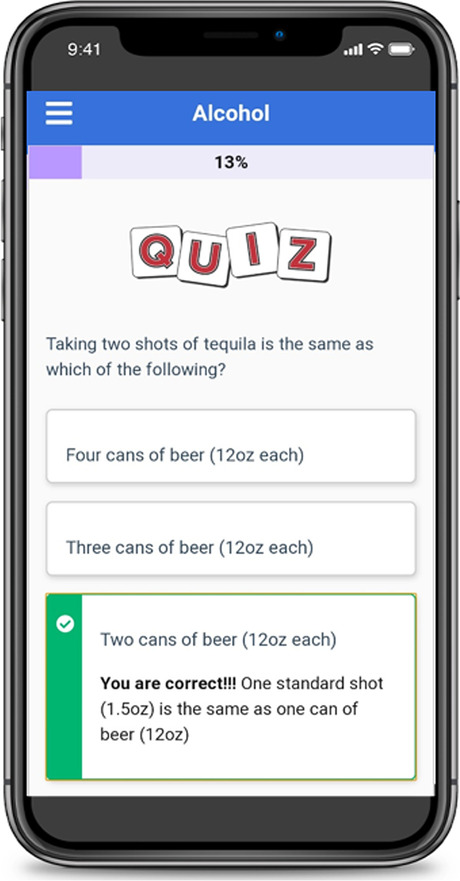
Example web-app quizzlet to test knowledge content

**Fig. 2:**
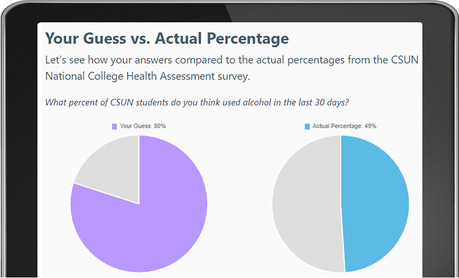
Example side-by-side pie charts to correct misperceptions

First, to address attitudes and knowledge, participants are introduced to each substance through educational facts, such as potency, identification, and effects on the brain and body. Providing a knowledge-based foundation at the beginning of each module supports the formation of positive attitudes regarding substance prevention and negative views of substance misuse and abuse [41, 49]. Next, to apply the construct of perceived susceptibility and correct misperceptions of risk, the module defines risky behavior and identifies potential risks of substance misuse. Additional content is provided on dependency, as well as identifying the health benefits of abstaining from substance use. Recognizing one’s risk for substance abuse and delineating related consequences reinforces intention to reduce or prevent risky substance use [40]. The web-app utilizes a variety of interactive approaches to address these behavior change strategies. For example, to facilitate dissemination of substance-specific knowledge, participants take quizzlets (see Fig. 1), flip content cards, and watch videos. The construct of subjective norms is addressed through normative re-education. Research demonstrates that adolescents and young adults commonly overestimate peer use; and a key prevention approach is to correct such misperceptions [42]. To practically apply this change strategy, the web-app asks participants to estimate the prevalence of peer substance use on campus, then view side-by-side pie charts to compare their “guess” to the “actual percentage” use as confirmed in the campus national college health assessment (See Fig. 2) [34].

The construct of self-efficacy is addressed through five evidence-based behavior change strategies (See Table 1) [39]. The first, personalized feedback, is applied as an in-app short self-assessment on how a participant’s (potential) substance use impacts daily life. In response, an algorithm tailors feedback and if appropriate, encourages students to initiate behavior change. Research demonstrates that strategies such as personalized feedback and self-assessment can significantly reduce substance use and help prevent future use [16, 43, 44]. Providing skills for responsible use, the second self-efficacy change strategy, is applied by highlighting the health rewards of reduced use (or non-use), and displaying responsible use checklists [50]. Refusal skills, the third self-efficacy change strategy, are addressed through examples of how to say “no” in a variety of scenarios. These refusal skills help empower one’s confidence to prevent or reduce substance use in social situations [47, 48], a common occurrence among many college students.

Additionally, each module includes protective strategies to increase self-efficacy. Flip cards with culturally and age-relevant images and language present healthier approaches to stress management and strategies to enhance wellbeing. These healthier approaches underscore the positive effects that strategies, such as physical activity and mindfulness, have on one’s physical, emotional, and mental health [51, 52]. To further demonstrate protective factors, culturally and age-relevant written content is provided on how to establish and strengthen positive social connections and seek out social support, a coping skill found to significantly aid young adults during challenging life events [53, 54]. To facilitate adoption and implementation of these protective behaviors, each module refers students to a web-app tab on the home screen that provides direct resources to access on-campus programs and activities.

Goal setting is the final evidence-based behavior change strategy used to address the construct of self-efficacy. The concluding interactive element of each module encourages participants to write a SMART goal statement to facilitate an action plan for changing risky substance behavior or sustaining responsible use (or non-use). Setting SMART goals (Specific, Measurable, Achievable, Realistic, and Timed) is a significant strategy frequently used in health behavior program planning and intervention development to provide participants criteria on how to create a behavior-specific goal with a well-defined measure for success [55, 56]. Each module provides an example SMART goal for participants to model, such as, “The next time I go out with friends I will order a mocktail instead of an alcoholic drink,” and enables participants to email the goal to themselves if they wish.

### Implementation and evaluation of the intervention

**Fig. 3:**
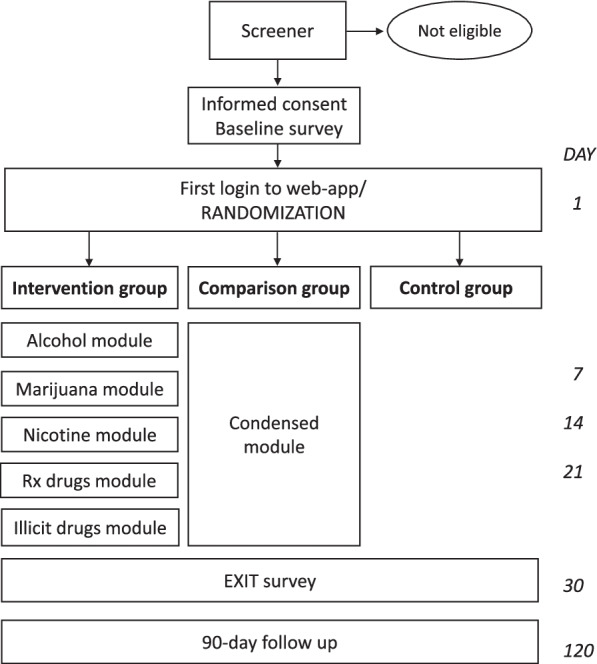
Schematic of study design

The 30-day iSTART web-app intervention is evaluated through a time series design. The study includes an eligibility screener, a baseline survey, a 30-day exit survey, and a 90-day follow-up survey (see Fig. 3: Schematic of study design). The web-app has been launched at a public university in southern California and is currently undergoing data collection for three years.

#### Eligibility

Interested students first complete a Qualtrics eligibility screener accessible via the study website. Matriculated students ages 18–30 who identify as low risk for AUD or SUD on the brief DSM-5 criteria screener are eligible to participate, and are redirected to the Qualtrics informed consent and baseline survey. Students who are screened as high risk are referred to additional resources for substance abuse treatment and excluded from the study. Those who agree to participate and complete the baseline survey are subsequently added to the web-app by study staff, and receive an automated welcome email with log-in instructions.

#### Randomization

The web-app is designed to randomize participants to intervention, comparison, or control groups. Upon first login, each participant is automatically randomized into one of the three conditions, and is provided access to the appropriate web-modules: the five weekly substance-specific modules for the intervention group, the single abbreviated module if in the comparison group, or no access to modules if in the control group. The web-application is also designed to keep track of completion dates, or non-completion, and send out automatic reminder emails and new module availability messages to participants.

#### Accessibility

Participants in the intervention and comparison groups can access the web-app on any device that connects to the internet (e.g., smartphone, tablet, laptop, computer). Students can log on using the web-app link embedded in the weekly module reminder email, or directly from the study website. Those in the intervention group have seven days to complete each substance-specific module, and upon completion receive the next module six days later. Those in the comparison group have 30 days to complete the single abbreviated substance module. All participants may contact study staff via email with questions or concerns, and have access to additional study information or support resources (e.g., helplines, treatment facilities, etc.) on the study website. To address any technical errors or server support, study staff communicate with university IT staff directly.

#### Incentives

Incentives are provided to ensure participation and retention. Participants receive Amazon.com gift cards via email when they complete pre- and post-test surveys and web-app modules. Exit surveys are auto-administered via email from the web-app. The follow-up survey is emailed by study staff to participants 90 days after exit surveys are completed. Participants have two weeks to complete exit and 90-day follow-up surveys. Those in the intervention group receive a total of $100**:** $15 for baseline, $15 for modules 1 and 2, $15 for modules 3 and 4, $25 for module 5 and the exit survey, and $30 for the 90-day follow-up survey. Comparison group participants receive a total of $75: $15 for baseline, $15 for the single module, $15 for the exit survey, and $30 for the 90-day follow-up survey. The control group receives a total of $65: $15 for baseline, $20 for the exit survey, and $30 for the 90-day follow-up survey.

### Measures

#### Primary outcome measures

Substance use behavior will be assessed at baseline, exit, and 90-day follow-up as required by SAMHSA’s Center for Substance Abuse Prevention (CSAP) Government Performance and Results Act (GPRA) adult questionnaire [57]. This includes a measure of frequency of self-reported past 30-day use for days drinking alcohol, binge drinking (as defined by five or more alcoholic beverages at the same time for males; four or more for females), use of various tobacco products (i.e., cigarettes, pipe tobacco, chewing tobacco, snus), electronic vaping, marijuana use, and non-prescribed prescription drug use (i.e., stimulants, sedatives, benzodiazepines, and prescription opioids). Additionally, past 30-day use of illicit drugs, including non-prescription opioids (i.e., cocaine, amphetamines, MDMA/Ecstasy, heroin, fentanyl, etc.) are assessed. Participants indicate the frequency of use on a continuous scale from ‘0’ to ‘30’ days.

Behavioral intent will be examined across all three time points with three items: intent to smoke marijuana/cigarettes, binge drink, and use non-prescribed drugs. Participants are asked to rate the likelihood of use in the next 30 days on a four-point Likert scale from 1 = v*ery likely to* 4 *= very unlikely*. A measure of substance use intention is necessary to determine the effectiveness of the web-app intervention on preventing or reducing participants’ intent to use; and is commonly used in behavior change theory research to determine actual use [58, 59].

#### Alcohol-specific outcomes

As the most frequently used substance among college students [60], ten measures will assess alcohol-related problems at baseline, exit and follow-up adapted from the Rutgers Alcohol Problem Index (RAPI) [61, 62]. Participants who report any past 30-day alcohol use indicate how many times they have experienced ten select outcomes in the last 30 days while drinking alcohol, or as the result of their alcohol use. Example items include: ‘Not being able to do your homework or study for a test’, ‘Missed out on other things because you spent too much money on alcohol’, ‘Went to work or school high or drunk’, ‘Felt that you needed more alcohol than you used to use in order to get the same effect’, and ‘Had withdrawal symptoms, that is, felt sick because you stopped or cut down on drinking’. Participants respond: *never, 1–2 times, 3–5 times, 6–10 times*, and *more than 10 times* in the past 30 days. Prior studies have demonstrated the ability of RAPI to detect DSM-5 AUDs among college students [63], and support its use in the present intervention to determine whether the web-app intervention reduces such negative outcomes at exit and 90-day follow-up compared to those in control and comparison groups.

#### Theoretical construct variables

In accordance with our theory constructs and behavior change strategies (see Table 1), assessment items also include psychosocial correlates that may predict intention to reduce or prevent substance use, such as knowledge, attitudes, risk perception, subjective norms, and self-efficacy*.*

**Attitudes/Knowledge**: To measure retention of substance-related knowledge in pre-, post-test and follow-up surveys, eight items related to potency, lawful use of substances, and risky or harmful use will be examined. These questions directly relate to information presented in the web-application and are intended to determine whether those in the intervention group learn and retain substance-related knowledge significantly more so than those in comparison group and the control group who may receive information in other settings or standardized prevention education on campus. Each item will be scored 1 = correct and 0 = incorrect, and a total knowledge score will be computed with a range between 0 and 8.

**Risk perceptions**: Participants will evaluate their perceived risk level across all three surveys as part of SAMHSA’s CSAP GPRA adult questionnaire [57], for the following six substance use behaviors: binge drinking once or twice a week, tobacco use once or twice a week, marijuana use once or twice a week, prescription opioid use once or twice a week, non-prescription opioid use once or twice a week, sharing needles/injection equipment, injecting drugs, and illicit drug use. Response options for each behavior include 1 = *no risk, 2 = slight risk, 3 = moderate risk, 4 = great risk, or 9 = don’t know.* This measure has been used in prior research to assess misperceptions of risk [64], and aligns with the construct of perceived susceptibility to identify risky behavior. Young adults who perceive themselves to be at moderate or high risk for harm from substance use are significantly less likely to report intention to use substances than those who report no or slight risk [64, 65]. Thus, providing college students with relevant, accurate, and reliable information about the risks and harms associated with substance use can significantly contribute to the prevention or reduction of such use.

**Subjective norms**: Normative re-education will be measured by assessing perceived prevalence of peer substance use at pre-, post-test, and follow-up. Participants indicate what percentage of students on campus they perceive use a variety of substances within the last 30 days, (i.e., alcohol, cigarettes, marijuana, different prescription drugs, and several illicit drugs) choosing one of ten percentile intervals from 0 to 100%. Prior research demonstrates that individuals who recognize peer use to be lower than originally perceived, are less likely to use substances when seeking peer approval [66, 67]. This measure is beneficial to examining the effectiveness of the behavior change strategy of normative re-education among those in the web-app intervention group, and whether such an approach significantly contributes to substance prevention over time.

**Self-efficacy***:* Self-efficacy will be evaluated through two refusal skills statements: “I would be able to say no if a friend offered me a drink of alcohol” and “I would be able to refuse if a friend offered me drugs, including marijuana” [68], by indicating their level of agreement on a four-point Likert scale from 1 = *strongly agree to 4* = *strongly disagree.* These measures are adapted from SAMHSA’s CSAP GPRA youth questionnaire, and are intended to determine whether participants exposed to the web-app report an increase in confidence to refuse substances over time more so than those in the comparison or control conditions. Prior studies suggest the inclusion of substance-related refusal skills measures are a beneficial tool in assessing self-efficacy, and such skills are effective in supporting the prevention or reduction of risky use [69, 70].

#### Other measurements for explorative research

**Socio-demographics***:* Participant characteristics, including age, Hispanic origin, race, sex at birth, gender identification, sexual orientation, living situation, college status, military service/veteran status, arrest history, parole/probation status, and household income will be assessed per SAMHSA’s GPRA adult questionnaire [57].

**Adverse Childhood Experiences (ACE):** At baseline, the ACE questionnaire will be used to measures ten traumatic or stressful childhood events experienced before the age of 18. ACE are conceptualized as maltreatment (e.g., verbal, emotional, physical, and sexual abuse), and household dysfunction (e.g., parent/caregiver substance use, intimate partner violence, incarceration, homelessness, mental illness, separation/divorce) [71]. Participants respond to each of the ten ACE items as 1 = Yes or 0 = No. An ACE score will be computed with a range from 0 to 10. Research has shown that ACE are among the most robust predictors of risky substance use behaviors, including early onset drinking and illicit drug use and prescription drug misuse among college students [72, 73] and that the negative consequences of ACE can vary by ethnicity and gender [74, 75].

**Developmental transition***:* Stressors typically experienced by young adults [76] and associated with substance use [77, 78] will also be explored. At baseline, participants respond 1 = Yes or 0 = No to a list of 18 major life events within the last three years. Example life events include: getting into a new romantic relationship; breaking up with a girlfriend or boyfriend; losing a job; being unemployed and not able to find work; caring for a parent or relative; getting extremely ill; having a baby; losing a baby; or having family separated due to immigration [76]. A total score will be computed with a range from 0 to 18.

### Data management and analysis

All data is collected online using Qualtrics, a fully HIPAA compliant software licensed by the university [79]. To assess program effects within prevention group students, we will calculate Cohen’s *d* to describe the magnitude and direction of the effect size. To examine program outcomes (e.g., alcohol, marijuana, and nicotine use etc.) over time, we will use statistical approaches that account for repeated measures nested within students, determine if longitudinal outcome trajectories are linear or quadratic and identify patterns of stability and change in outcomes over the study period using appropriate modeling approaches. Prevention, comparison, and control students will participate for three waves of data collection. To test whether prevention app participants have no or slower rates of growth in substance use relative to comparison or control students, two level models will estimate two regression equations simultaneously [a within-person equation (i.e., time-level model) and a between-person equation]. This will determine whether the slopes of substance use are less steep among prevention participants than comparison and control group students.

Generalized linear models will also test hypotheses that growth in mediators, for example increases in *knowledge*, will predict flat or slower increases in *substance use*, by including *knowledge* as a time-varying variable in the within-person model. Including individuals’ mean *knowledge* in the between-person intercept equation, and by group-mean centering the *knowledge* within the time-level equations, analyses separate out between-person differences, allowing for an assessment of within-student change in *knowledge* and its relation to *substance use* within the study period. This analytic approach can account for stable, between-student differences and potential bias associated with unmeasured, between-person factors that influence outcome behaviors [80, 81].

## Discussion

Despite effective strategies to prevent substance use and its disorders, evidence-based practices are not being broadly adapted and sustained. Coupled with emerging trends in opioids and vaping [28–30], the demand for innovative substance prevention research and practice among college students is imperative. The novel iSTART web-app intervention attempts to address limitations of current substance prevention programs on college campuses, as these programs primarily target alcohol abuse, rarely utilize mHealth platforms [17], and seldom explore substance prevention strategies among predominately Hispanic students [23]. The iSTART interactive web-app was designed to appeal to college students’ use of smart devices, as well as their preference for and comfortability with web-based technology. The web-app aims to use five interactive modules to engage and inform students at a Hispanic Serving Institution to prevent risky substance use (i.e., alcohol, marijuana, nicotine, prescription drugs, and illicit drugs).

During the development of the iSTART web-app, multiple strategies of intervention mapping were utilized, and numerous stakeholders were conferred. By including evidence-based theoretical constructs, and individual-level behavior change strategies that are practically applied and targeted toward substance use prevention among college students, we believe the iSTART web-app is an innovative and sustainable program ideal for college campuses with diverse student populations. If this prevention web-app is successful, it will significantly contribute to the evidence of effective substance use interventions in the college setting, and identify the benefits of web-app programs to prevent future substance use.

## Data Availability

Not applicable.

## Abbreviations

HABLAS: Hispanic Americans Baseline Alcohol Survey
NIAAA: National Institute on Alcohol Abuse and Alcoholism
SAMHSA: Substance Abuse and Mental Health Services Administration
BMI: Brief motivational interventions
BASICS: Brief Alcohol Screening and Intervention for College Students
HP: Healthy People
iSTART: Initiative for Services in Tech-Health and Rapid Testing
CSUN: California State University, Northridge
AUD: Alcohol use disorder
SUD: Substance use disorder
DSM-5: The Diagnostic and Statistical Manual of Mental Disorders, Fifth Edition
NCHA: National College Health Assessment
CBO: Community-based organization
ATOD: Alcohol, tobacco, and other drugs
CDC: Centers for Disease Control and Prevention
NIDA: National Institutes of Drug Abuse
FDA: U.S. Food and Drug Administration
CSAP: Center for Substance Abuse Prevention
GPRA: Government Performance and Results Act
RAPI: Rutgers Alcohol Problem Index
ACE: Adverse Childhood Experiences

## References

[1] American college health Association (2020). National College Health Assessment III: reference group executive summary fall 2020.

[2] Arnett JJ (2000). Optimistic bias in adolescent and adult smokers and nonsmokers. Addict Behav.

[3] Hingson R, White A (2014). New research findings since the 2007 surgeon General’s call to action to prevent and reduce underage drinking: a review. J Stud Alcohol Drugs.

[4] Schwartz SJ, Côté JE, Arnett JJ (2005). Identity and agency in emerging adulthood: two developmental routes in the individualization process. Youth Soc.

[5] Buckles K, Hagemann A, Malamud O, Morrill M, Wozniak A (2016). The effect of college education on mortality. J Health Econ.

[6] Schafer MH, Wilkinson LR, Ferraro KF (2013). Childhood (mis) fortune, educational attainment, and adult health: contingent benefits of a college degree?. Soc Forces.

[7] Hingson RW, Zha W, Weitzman ER (2009). Magnitude of and Trends in Alcohol-Related Mortality and Morbidity Among U.S. College Students Ages 18–24, 1998–2005. J Stud Alcohol Drugs.

[8] Presley CA, Pimentel ER (2006). The introduction of the heavy and frequent drinker: a proposed classification to increase accuracy of alcohol assessments in postsecondary educational settings. J Stud Alcohol.

[9] Jones CM, Clayton HB, Deputy NP, Roehler DR, Ko JY, Esser MB (2020). Prescription Opioid Misuse and Use of Alcohol and Other Substances Among High School Students — Youth Risk Behavior Survey, United States, 2019. MMWR Suppl.

[10] Egan KL, Reboussin BA, Blocker JN, Wolfson M, Sutfin EL (2013). Simultaneous use of non-medical ADHD prescription stimulants and alcohol among undergraduate students. Drug Alcohol Depend.

[11] National Institute on Alcohol Abuse and Alcoholism. College Drinking. Bethesda (MD): National Institute on Alcohol Abuse and Alcoholism; October 2021. Available from: https://www.niaaa.nih.gov/publications/brochures-and-fact-sheets/college-drinking.

[12] Chartier K, Caetano R (2010). Ethnicity and health disparities in alcohol research. Alcohol Res Health.

[13] Vaeth PA, Caetano R, Ramisetty-Mikler S, Rodriguez LA (2009). Hispanic Americans baseline alcohol survey (HABLAS): alcohol-related problems across Hispanic national groups. J Stud Alcohol Drugs.

[14] Substance Abuse and Mental Health Services Administration. The Opioid Crisis and the Hispanic/Latino Population: An Urgent Issue. No. PEP20-05-02-002. Bethesda (MD): U.S. Department of Health and Human Services, Office of Behavioral Equity; 2020. Available from: https://store.samhsa.gov/sites/default/files/SAMHSA_Digital_Download/PEP20-05-02-002.pdf.

[15] Substance Abuse and Mental Health Services Administration. 2019 National Survey on Drug Use and Health: Hispanics 2020. Rockville (MD): U.S. Department of Health and Human Services; Nov 2020. Available from: https://www.samhsa.gov/data/report/2019-nsduh-hispanics-latino-or-spanish-origin-or-desce.

[16] National Institute on Alcohol Abuse and Alcoholism. CollegeAIM Alcohol Intervention Matrix: Individual-Level Strategies. Rockville (MD): National Institute on Alcohol Abuse and Alcoholism; 2019. Available from: https://www.collegedrinkingprevention.gov/CollegeAIM/IndividualStrategies/default.aspx.

[17] Prevention Solutions @ Education Development Center. Preventing Substance Misuse Among 18-25 Year Olds: Program and Strategies. Rockville (MD): Substance Abuse and Mental Health Services Administration’s Center for the Application of Prevention Technologies Task Force; April 2018. Available from: https://preventionsolutions.edc.org/sites/default/files/attachments/Preventing-Substance-Misuse-18-25-Year-Olds-Programs-and-Strategies_0_0.pdf.

[18] Saltz RF (2004). Preventing alcohol-related problems on college campuses: summary of the final report of the NIAAA task force on college drinking. Alcohol Res Health.

[19] Blueprints for Healthy Youth Development. Brief Alcohol Screening and Intervention for College Students (BASICS). Boulder (CO): University of Colorado Boulder; 2021. Available from: https://www.blueprintsprograms.org/programs/203999999/brief-alcohol-screening-and-intervention-for-collegestudents-basics/.

[20] Prosser T, Gee KA, Jones F (2018). A meta-analysis of effectiveness of E-interventions to reduce alcohol consumption in college and university students. J Am Coll Heal.

[21] Paschall MJ, Antin T, Ringwalt CL, Saltz RF (2011). Evaluation of an internet-based alcohol misuse prevention course for college freshmen: findings of a randomized multi-campus trial. Am J Prev Med.

[22] Mitchell MM, Mendelson J, Gryczynski J, Carswell SB, Schwartz RP (2020). A novel telehealth platform for alcohol use disorder treatment: preliminary evidence of reductions in drinking. Am J Drug Alcohol Abuse.

[23] Tuckson RV, Edmunds M, Hodgkins ML (2017). Telehealth. N Engl J Med.

[24] Hennessy EA, Tanner-Smith EE, Mavridis D, Grant SP (2019). Comparative effectiveness of brief alcohol interventions for college students: results from a network meta-analysis. Prev Sci.

[25] Larimer ME, Cronce JM, Lee CM, Kilmer JR (2004). Brief intervention in college settings. Alcohol Res Health.

[26] Ickes MJ, Haider T, Sharma M (2015). Alcohol abuse prevention programs in college students. J Subst Abus.

[27] Plotnikoff RC, Costigan SA, Kennedy SG, Robards SL, Germov J, Wild C (2019). Efficacy of interventions targeting alcohol, drug and smoking behaviors in university and college students: a review of randomized controlled trials. J Am Coll Heal.

[28] Throckmorton DC, Gottlieb S, Woodcock J (2018). The FDA and the next wave of drug abuse—proactive pharmacovigilance. N Engl J Med.

[29] Schulenberg J, Johnston L, O’Malley P, Bachman J, Miech R, Patrick M. Monitoring the Future National Survey results on Drug Use, 1975-2019: Volume II, College Students and Adults ages 19–60. Ann Arbor (MI): Institute for Social Research, University of Michigan; 2020. Available from: https://deepblue.lib.umich.edu/bitstream/handle/2027.42/162576/2019-20%20VOL%20II%20FINAL%203.pdf?sequence=1.

[30] Compton WM, Jones CM, Baldwin GT, Harding FM, Blanco C, Wargo EM (2019). Targeting youth to prevent later substance use disorder: an underutilized response to the US opioid crisis. Am J Public Health.

[31] National Center for Education Statistics. Number and Percentage of Students Enrolled in Degree-Granting Postsecondary Institutions, by Distance, Education, Participation, Location of Student, Level of Enrollment, and Control and Level of Institution: Fall 2015 and Fall 2016. Washington (DC): U.S. Department of Education; January 2018. Available from: https://nces.ed.gov/programs/digest/d17/tables/dt17_311.15.asp.

[32] Healthy People 2030. Drug and Alcohol Use. Washington (DC): Department of Health and Human Services; August 2020. Available from: https://health.gov/healthypeople/objectives-and-data/browse-objectives/drug-and-alcohol-use.

[33] Eldredge LKB, Markham CM, Ruiter RAC, Fernández ME, Kok G, Parcel GS (2016). Planning health promotion programs : an intervention mapping approach.

[34] American College Health Association (2018). National College Health Assessment II: California State University Northridge executive summary spring 2018.

[35] Rainisch B, Forster M, Karamehic N, Cornejo M, Dahlman L. Pilot of a telehealth brief alcohol intervention for college students at a Hispanic serving institution. J Am Coll Heal. 2022:1–7. 10.1080/07448481.2022.2054278. Published online: 29 Mar 2022. 10.1080/07448481.2022.2054278 35348433

[36] Health Research for Action UC Berkeley. Healthy Communication Tips. Berkeley (CA): University of California, Berkeley; 2021. Available from:http://www.healthresearchforaction.org/sites/default/files/HRA%20Health%20Communication%20Tips_0.pdf.

[37] Generation Rx. University: Resources for College Students. Columbus (OH): The Ohio State University, College of Pharmacy; 2020. Available from: https://generationrx.org/toolkits/university/.

[38] ReachOut.com. Pyrmont (AU): ReachOut Australia; 2022. Available from: https://au.reachout.com.

[39] Kadden RM, Litt MD (2011). The role of self-efficacy in the treatment of substance use disorders. Addict Behav.

[40] Hughes A, Lipari RN, Williams MR. Marijuana Use and Perceived Risk of Harm from Marijuana Use Varies Within and Across States, The CBHSQ Report. Rockville (MD): Substance Abuse and Mental Health Services Administration; 2016. Available from: https://www.ncbi.nlm.nih.gov/books/NBK396156/pdf/Bookshelf_NBK396156.pdf. 27854415

[41] Cooke R, Dahdah M, Norman P, French DP (2016). How well does the theory of planned behaviour predict alcohol consumption? A systematic review and meta-analysis. Health Psychol Rev.

[42] Walters ST, Vader AM, Harris TR (2007). A controlled trial of web-based feedback for heavy drinking college students. Prev Sci.

[43] Saitz R, Palfai TP, Freedner N, Winter MR, Macdonald A, Lu J, Ozonoff AL, Rosenbloom DL, Dejong W (2007). Screening and brief intervention online for college students: the ihealth study. Alcohol Alcohol.

[44] Ganz T, Braun M, Laging M, Schermelleh-Engel K, Michalak J, Heidenreich T (2018). Effects of a stand-alone web-based electronic screening and brief intervention targeting alcohol use in university students of legal drinking age: a randomized controlled trial. Addict Behav.

[45] Hagger MS, Lonsdale A, Chatzisarantis NLD (2012). A theory-based intervention to reduce alcohol drinking in excess of guideline limits among undergraduate students.

[46] Kenney SR, Napper LE, LaBrie JW, Martens MP (2014). Examining the efficacy of a brief group protective behavioral strategies skills training alcohol intervention with college women. Psychol Addict Behav.

[47] Onrust SA, Otten R, Lammers J, Smit F (2016). School-based programmes to reduce and prevent substance use in different age groups: what works for whom? Systematic review and meta-regression analysis. Clin Psychol Rev.

[48] Witkiewitz K, Donovan DM, Hartzler B (2012). Drink refusal training as part of a combined behavioral intervention: effectiveness and mechanisms of change. J Consult Clin Psychol.

[49] Heckman CJ, Dykstra JL, Collins BN (2011). Substance-related knowledge, attitude, and behaviour among college students: opportunities for health education. Health Educ J.

[50] National Institute on Alcohol Abuse and Alcoholism. Rethinking Drinking: Alcohol your Heath. Bethesda (MD): National Institute on Alcohol Abuse and Alcoholism 2021. Available from: https://www.niaaa.nih.gov/sites/default/files/publications/NIAAA_RethinkingDrinking.pdf.

[51] Bodenlos JS, Noonan M, Wells SY (2013). Mindfulness and alcohol problems in college students: the mediating effects of stress. J Am Coll Heal.

[52] Prevention Plus Wellness. Lifestyle Behavior Programs Preventing Substance Use & Promoting Mental Health Augustine (FL): Prevention Plus Wellness; 2022. Available from: https://preventionpluswellness.com.

[53] Lee CY, Dik BJ (2017). Associations among stress, gender, sources of social support, and health in emerging adults. Stress Health.

[54] Kazemi DM, Borsari B, Levine MJ, Lamberson KA, Dooley B (2018). REMIT: development of a mHealth theory-based intervention to decrease heavy episodic drinking among college students. Addict Res Theory.

[55] McKenzie J, Neiger B, Thackeray R (2019). Health promotion programs.

[56] Bailey RR (2019). Goal setting and action planning for health behavior change. Am J Lifestyle Med.

[57] Substance Abuse and Mental Health Services Administration. National Minority AIDS Initiative (MAI) Substance Abuse/HIV Prevention Initiative-Adult Questionnaire. Rockville (MD); U.S. Department of Health and Human Services; 2021. Available from: https://www.samhsa.gov/sites/default/files/maimrt-new-adult-questionnaire.pdf.

[58] Wolford C, Swisher JD (1986). Behavioral intention as an indicator of drug and alcohol use. J Drug Educ.

[59] Montano DE, Kasprzyk D, Glanz K, Rimer B, Viswanath K (2015). Theory of reasoned action, theory of planned behavior, and the integrated behavioral model. Health behavior: theory, research and practice.

[60] National Institute on Drug Abuse. Drug and Alcohol Use in College-Age Adults in 2018. Baltimore (MD); National Institutes of Health; Sep 13, 2019. Available from: https://nida.nih.gov/drug-topics/trends-statistics/infographics/drug-alcohol-use-incollege-age-adults-in-2018.

[61] White HR, Labouvie EW . Rutgers Alcohol Problem Index (RAPI). Piscataway (NJ); Center of Alcohol Studies, Rutgers University; 1989. Available from: https://pubs.niaaa.nih.gov/publications/assessingalcohol/InstrumentPDFs/57_RAPI.pdf.

[62] White HR, Labouvie EW (1989). Towards the assessment of adolescent problem drinking. J Stud Alcohol.

[63] Hagman BT (2017). Diagnostic performance of the rutgers alcohol problem index (RAPI) in detecting DSM-5 alcohol use disorders among college students. J Addict Prev.

[64] Lipari RN, Jean-Francois B. Trends in Perception of Risk and Availability of Substance Use Among Full-Time College Students, The CBHSQ Report. Rockville (MD): Substance Abuse and Mental Health Services Administration; August 16, 2016. Available from: https://www.samhsa.gov/data/sites/default/files/report_2418/ShortReport-2418.pdf. 27854410

[65] Johnston L, O'Malley P, Bachman J, Schulenberg J (2012). Monitoring the future national survey results on drug use, 1975-2011: volume I, secondary school students.

[66] Larimer ME, Cronce JM (2002). Identification, prevention and treatment: a review of individual-focused strategies to reduce problematic alcohol consumption by college students. J Stud Alcohol Suppl.

[67] Benton SL, Downey RG, Glider PJ, Benton SA. College Students’ Norm Perception Predicts Reported Use of Protective Behavioral Strategies for Alcohol Consumption. J Stud Alcohol Drugs. 2008;69(6):859-65. 10.15288/jsad.2008.69.859. 10.15288/jsad.2008.69.859 18925344

[68] Substance Abuse and Mental Health Services Administration. National Minority AIDS Initiative (MAI) Substance Abuse/HIV Prevention Initiative-Youth Questionnaire. Rockville (MD): U.S. Department of Health and Human Services; 2021. Available from: https://www.samhsa.gov/sites/default/files/maimrt-new-youth-questionnaire.pdf.

[69] Young RM, Hasking PA, Oei TP, Loveday W (2007). Validation of the drinking refusal self-efficacy questionnaire—revised in an adolescent sample (DRSEQ-RA). Addict Behav.

[70] Foster DW, Neighbors C, Young CM (2014). Drink refusal self-efficacy and implicit drinking identity: an evaluation of moderators of the relationship between self-awareness and drinking behavior. Addict Behav.

[71] Felitti VJ, Anda RF, Nordenberg D, Williamson DF (1998). Adverse childhood experiences and health outcomes in adults: the ace study. J Fam Consum Sci.

[72] Forster M, Grigsby TJ, Rogers CJ, Benjamin SM (2018). The relationship between family-based adverse childhood experiences and substance use behaviors among a diverse sample of college students. Addict Behav.

[73] Krinner LM, Warren-Findlow J, Bowling J. Examining the Role of Childhood Adversity on Excess Alcohol Intake and Tobacco Exposure Among US College Students. Subst Use Misuse. 2020;55(13):2087–2098. 10.1080/10826084.2020.1790009. 10.1080/10826084.2020.1790009 32657199

[74] Forster M, Rogers CJ, Benjamin SM, Grigsby T, Lust K, Eisenberg ME (2019). Adverse childhood experiences, ethnicity, and substance use among college students: findings from a two-state sample. Subst Use Misuse.

[75] Grigsby TJ, Rogers CJ, Albers LD, Benjamin SM, Lust K, Eisenberg ME, Forster M (2020). Adverse childhood experiences and health indicators in a young adult, college student sample: differences by gender. Int J Behav Med.

[76] Arnett JJ (1998). Risk behavior and family role transitions during the twenties. J Youth Adolesc.

[77] Allem JP, Sussman S, Soto DW, Baezconde-Garbanati L, Unger JB (2016). Role transitions and substance use among Hispanic emerging adults: a longitudinal study using coarsened exact matching. Addict Behav.

[78] Forster M, Vetrone S, Grigsby TJ, Rogers C, Unger JB (2020). The relationships between emerging adult transition themes, adverse childhood experiences, and substance use patterns among a community cohort of Hispanics. Cult Divers Ethn Minor Psychol.

[79] Qualtrics. Provo (UT): Qualtrics; 2022. Available from: https://www.qualtrics.com.

[80] Raudenbush SW, Bryk AS (2002). Hierarchical linear models: applications and data analysis methods.

[81] Šidák Z (1967). Rectangular confidence regions for the means of multivariate normal distributions. J Am Stat Assoc.

